# Finite Element Simulation of NiTi Umbrella-Shaped Implant Used on Femoral Head under Different Loadings

**DOI:** 10.3390/bioengineering4010023

**Published:** 2017-03-12

**Authors:** Reza Mehrabi, Milad Dorri, Mohammad Elahinia

**Affiliations:** 1 Department of Mechanical Engineering, Vali-e-Asr University of Rafsanjan, Rafsanjan 77139-36417, Iran; milad.dorri@yahoo.com; 2 Dynamic and Smart Systems Laboratory, MIME Department, University of Toledo, Toledo, OH 43606, USA; mohammad.elahinia@utoledo.edu

**Keywords:** shape memory alloy, umbrella-shaped, numerical model, femoral head, implant

## Abstract

In this study, an umbrella-shaped device that is used for osteonecrosis treatment is simulated. The femoral head is subjected to various complex loadings as a result of a person’s daily movements. Implant devices used in the body are made of shape memory alloy materials because of their remarkable resistance to wear and corrosion, good biocompatibility, and variable mechanical properties. Since this NiTi umbrella-shaped implant is simultaneously under several loadings, a 3-D model of shape memory alloy is utilized to investigate the behavior of the implant under different conditions. Shape memory and pseudo-elasticity behavior of NiTi is analyzed using a numerical model. The simulation is performed within different temperatures and in an isothermal condition with varied and complex loadings. The objective of this study is to evaluate the performance of the device under thermal and multi-axial forces via numerically study. Under tensile loading, the most critical points are on the top part of the implant. It is also shown that changes in temperature have a minor effect on the Von Mises stress. Applied forces and torques have significant influence on the femoral head. Simulations results indicate that the top portion of the umbrella is under the most stress when embedded in the body. Consequently, the middle, curved portion of the umbrella is under the least amount of stress.

## 1. Introduction

**Figure 1:**
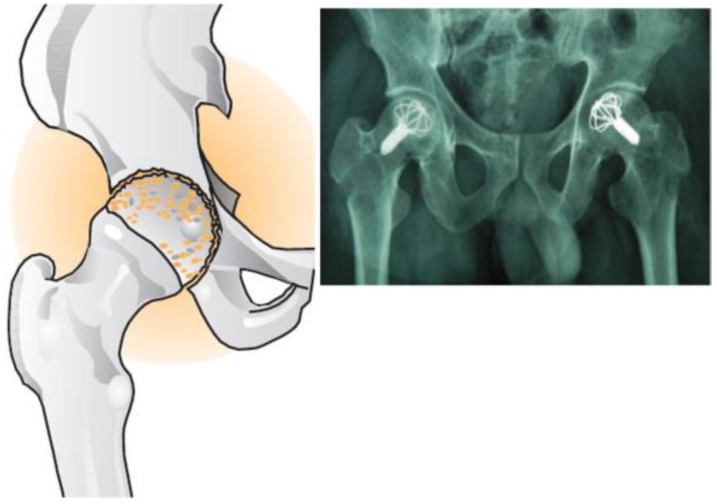
Umbrella-shaped implant used in the femoral head [1]**.**

Osteonecrosis is a dangerous disease appearing in young patients aged between 30 to 40 years [1]. If this disease is not effectively treated, it leads to femoral head decay and will cause the femoral head to collapse (Figure 1). To stop decay, an umbrella-shaped device is implanted in the femoral head. Recently, an umbrella-shaped device made of shape memory alloy (NiTi) has been a suggested replacement for the femoral head [1]. Nitinol’s (NiTi) strength, biocompatibility, remarkable resistance to wear and corrosion, anti-decay, shape memory, and pseudo-elastic behavior has a distinct position in medicine. According to 17 devices implanted in 10 patients, the efficiency rate is 82.35% [1]. Christ et al. [2] worked on the finite element model for SMA considering thermomechanical couplings in large strain. The effect of pseudo-elasticity and shape memory effect (SME) was the objective of their study. Saleeb et al. carried out a study on the importance of shape memory materials; they assessed performance characteristics and clinical forces in simulated shape memory bone surgical procedures [3]. The compressive force was developed in the bone staple and the simulation results positively corresponded with the experimental results. The body temperature-activated bone staple proved to be clinically viable and provided a staple clamping force needed for speedy cooptation of the broken bones. Brown et al. [4] studied the mechanical characteristics of bone in femoral aseptic necrosis. Unique differences in the revascularization occurring within femoral heads lead to significant variability in strength and stiffness. The tested samples show the significant reduction in yield strength, reducing elastic modulus, and increase the strain to failure in comparison with samples from normal femoral heads. The quality of life following femoral osteotomy and total hip arthroplasty for nontraumatic osteonecrosis of the femoral head was studied by Seki and his colleagues [5]. They showed physical disorder is a more powerful factor than pain for reducing the quality of life in the nonoperative group compared to the surgical group. In addition, osteotomy and total hip arthroplasty were similar on the assessment after surgery of quality of life score if the indications for osteotomy were strictly applied. Floerkemeier and his colleagues analyzed core decompression and osteonecrosis intervention rod in osteonecrosis of the femoral head using clinical outcomes and finite element analysis [6]. The FEM results with clinical and MRI results were confirmed. The risk of fracture during extreme loading was confirmed. The core decompression using small drill holes is superior compared to the conventional core decompression and tantalum implant. Yu et al. in order to estimate the efficiency rate of the umbrella-shaped piece, implanted the umbrella shape in 10 patients and the results were satisfactory [1]. Mechanical behavior of umbrella-shaped NiTi femoral head during implant operation was studied by Yi et al. [7]. In their work, femoral head and an umbrella-shaped implant were investigated by the finite element method. They investigated how the ultimate shapes of the support device functioned in the human body.

Today’s newly developed smart materials are beneficial within different industrial sectors, as these materials have one or more properties and respond to external conditions. For example, if the material is subjected to an electric field, its properties change as a piezoelectric. Other examples of smart materials include shape memory alloys (SMAs) possessing variable mechanical properties at different conditions. These alloys are used in devices that can be returned to the original shape under a certain stress or temperature. They have two stable phases, called austenite and martensite, capable of transformation. Smart material alloys are also used in industries like aerospace, medicine, car manufacturing, and robotics.

The first continuing studies of shape memory alloys are attributed to Buehler and his colleagues in 1961 [8]. Their studies were focused mainly on nickel-titanium alloy. Liang and Rogers developed one-dimension thermomechanical constitutive model for shape memory alloy [9]. In their research, they studied thermomechanical model formulation, matter properties such as internal phase transformation, and temperature-stress-strain relation. Brinson studied the constitutive behavior of one-dimensioned shape memory alloys [10]. She defined non-constant material functions and a new definition of the martensitic variable. Brinson and Huang in 1996 developed an SMA structural model and compared it with other works [11]. Auricchio developed a one-dimensioned model for shape memory alloys with different elastic properties between austenite and martensite and extended the model for simulation of biomedical devices [12]. However, all these models were applied for one-dimensional states, not for three-dimensional applications. To solve this problem, Boyd and Lagoudas developed a three-dimensional model [13]. Similarly, Lim and McDowell analyzed a three-dimensional model for multi-axial proportional and nonproportional loading [14]. Recently, Mehrabi et al. presented a three-dimensional phenomenological model based on microplane theory for SMAs [15]. They generalized 1-D equations to develop 3-D equations. In addition, they studied the constitutive model of tension-torsion coupling and tension-compression asymmetry to simulate the behavior of shape memory alloys [16,17]. They investigated the effect of boundary condition on the mechanical behavior of NiTi [18]. Here, the constitutive model proposed by authors is used to simulate a real device under complex conditions.

In the present study, an umbrella-shaped device made of Nitinol that was proposed by Yi et al. [7] is numerically simulated here. To this end, the 3-D model that has been developed is utilized to investigate the effect of different temperatures on the umbrella-shaped implant. Here, SMA behavior under different forces, torques, and temperatures are investigated. The critical points on the implant are first qualitatively compared with Yi et al. study [7], and then numerical results are extended to the different conditions. High and low temperature as well as tension and torsion loadings are some of the differences between the current paper and Yi study. Stress-strain behavior, the effect of force and torque on the critical points, and performance of the implant are investigated.

## 2. Modeling and Simulation 

**Figure 2:**
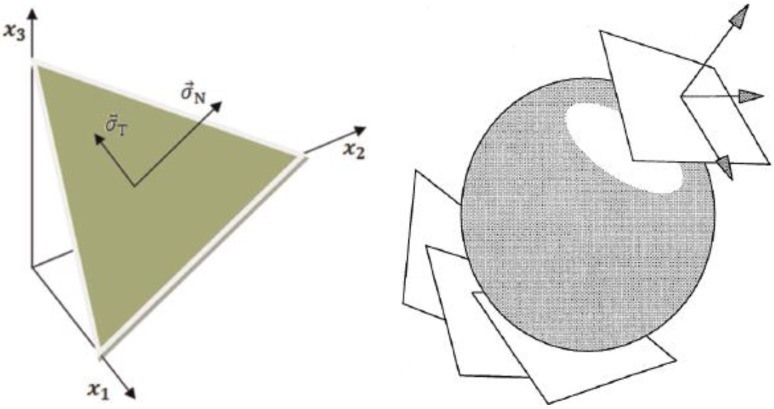
Stress components on a microplane.

The SMA microplane model considers the possibility of martensitic transformation on several planes with different orientations. It generally obtains the transformation strain as a superposition of normal-shear-induced transformation strains to reproduce the actual physical behavior of SMAs. In this method, the stress vector on each microplane is related to the macroscopic stress tensor. Figure 2 shows the normal stress vector and shear stress vector on a microplane. If the normal vector on a microplane at a given point is demonstrated as n, the macroscopic stress tensor to achieve normal stress vector on the same plane demonstrated by σ. Therefore, $\sigma_{N}$, is described as follow:

(1) $$\sigma_{N}=N_{ij}\sigma_{ij}$$

tensor *N* is defined based on factors of normal vector components as follow:

(2) $$N_{ij}=n_{i}n_{j}$$

Stress compositions can be written as is shown in Figure 2. Shear stress component on a microplane is:

(3) $$\sigma_{T}=T:\sigma$$

the tensor *T* has the Cartesian components as:

(4) $$T_{ij}=\frac{\left(n_{i}t_{j}+n_{j}t_{i}\right)}{2},\;t_{i}=\frac{\sigma_{ik}n_{k}-\sigma_{N}n_{i}}{\sqrt{\sigma_{jr}\sigma_{js}n_{r}n_{s}-\sigma_{N}^{2}}}$$

in which $n_{i}$ represents the components of the unite normal vector (*n*) on the plane. Based on the principle of complementary virtual work between macroscopic and quantity defined in the microplane through each point, the following equation is established:

(5) $$\int_{\varphi }\varepsilon :\delta \sigma d\varphi =\int_{\varphi }\left(\varepsilon_{N}\delta \sigma_{N}+\varepsilon_{T}\delta \sigma_{T}\right)d\varphi$$

where φ is one surface of a sphere. As shown in Figure 2, in order to consider all the surfaces passing through a point, the complementary virtual work is expressed on the sphere at that point. For more details about formulation, refer to reference [16]. Microplane tangential strain consists of tangential elastic strain and tangential transformation strain.

(6) $$\varepsilon_{T}=\varepsilon_{T}^{e}+\varepsilon_{T}^{tr}$$

Tangential transformation strain can be obtained from the following equation:

(7) $$\varepsilon_{T}^{tr}=\varepsilon^{*}\xi \left(\bar{\sigma },T\right)$$

where $\varepsilon^{*}$ is the axial maximum recoverable strain and $\xi \left(\bar{\sigma },T\right)$ is the martensite volume fraction as a function of the effective stress, $\bar{\sigma }$, and temperature, *T*. In the present study, effective stress can be expressed as:

(8) $$\bar{\sigma }=\sqrt{\sigma^{2}+3\tau^{2}}$$

where σ is the macroscopic tensile stress and τ is the macroscopic shear stress. For $\xi \left(\bar{\sigma },T\right)$, equations are used which were proposed by Brinson (1993).

Using the method described in the reference [15], the elastic and transformation strains are derived as:

(9) $$\varepsilon_{ij}=\varepsilon_{ij}^{e}+\varepsilon_{ij}^{tr}$$

(10) $$\varepsilon_{ij}^{e}=-\frac{\upsilon }{E\left(\xi \right)}\sigma_{ss}\delta_{ij}+\frac{1+\upsilon }{E\left(\xi \right)}\sigma_{rs}\cdot \frac{3}{4\pi }\underset{\varphi }{\int }\left(N_{rs}N_{ij}+T_{rs}T_{ij}\right)d\varphi$$

(11) $$\varepsilon_{ij}^{tr}=\varepsilon^{*}\cdot \xi \left(\bar{\sigma },T\right)\cdot \frac{3}{4\pi }\underset{\varphi }{\int }T_{ij}d\varphi$$

In the calculation of the strain, the above integrals are evaluated numerically by using a 42-point Gaussian integration formula for a sphere surface (Bazant and Oh, 1986) [19].

In this study, the above-mentioned constitutive model is utilized and material parameters for NiTi are calibrated. Table 1 shows the mechanical properties of NiTi alloy which are extracted from references [7,10,18]. The simulation was performed by ABAQUS software version 6.14. In the first step, the umbrella-shaped implant is placed under tension. In the second step, the implant is placed under torsion and in the third step; it is placed under both tension-torsion loading. In each step, one simulation at a temperature above $A_{f}$ and another one at temperature lower $A_{s}$ are being analyzed. Table 1 shows that constants $C_{M}$ and $C_{A}$ are material properties in temperature-stress phase diagram. $E_{a}$ and $E_{m}$ are respectively austenite elastic module and martensite elastic module. $M_{f}$, $M_{s}$, $A_{s}$, $A_{f}$, $\sigma_{s}^{\mathrm{cr}}$ and $\sigma_{f}^{\mathrm{cr}}$ are martensite final temperature, martensite start temperature, austenite start temperature, austenite final temperature, start critical stress, and the final critical stress, respectively.

**Figure 3:**
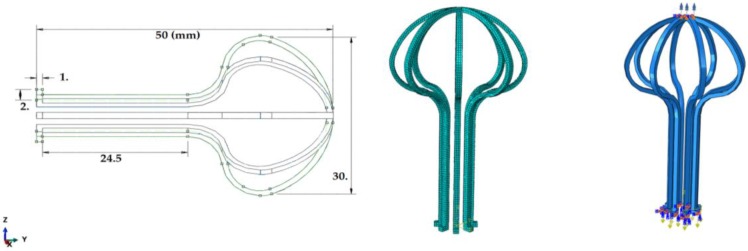
Umbrella-shaped implant sizes, mesh, and boundary conditions (left to right).

Developed subroutine [16] is linked to Abaqus software to analyze the implant. In this simulation, the umbrella-shaped implant length is 50 mm; the umbrella outer diameter is 30 mm; the thickness is 1 mm; and the base bent is 2 mm (Figure 3) [1,7]. After the mesh study, the element type of C3D8R was chosen and applied. For the boundary conditions, the upper part of the piece is fixed and its legs are placed under different uniaxial tensions, torsions, multiaxial tension-torsion forces, and thermal loading. In this process, loading and then unloading were applied. Figure 3 shows boundary conditions and mesh size for the umbrella-shaped implant. It is under complex loadings, so it needs to have a high degree of flexibility. Tension is applied on the bottom with green lines while torsion is applied on the top of the implant as shown in Figure 3.

## 3. Results and Discussion

### 3.1. Study of the Behavior of Umbrella-Shaped Implant at Temperature Above $A_{f}$

#### 3.1.1. Pseudoelastic Behavior under Simple Tension

**Figure 4:**
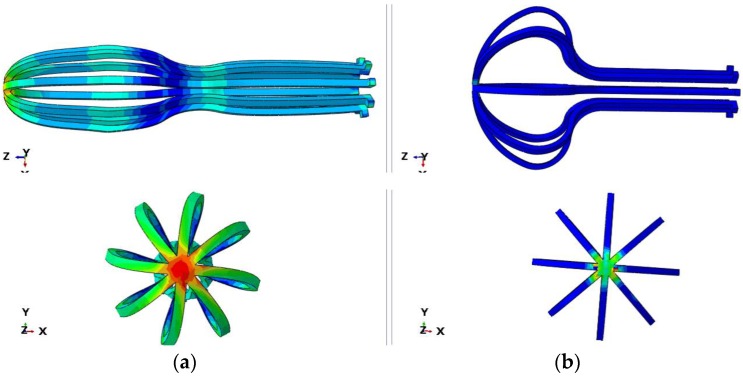
Umbrella-shaped implant under loading (**a**) and unloading (**b**).

The femoral head undergoes complex forces during walking. For example, a person who is exercising can impose different stretch forces on their femoral head. The implant must be flexible enough to return to its initial shape after each loading and unloading (Figure 4).

**Figure 5:**
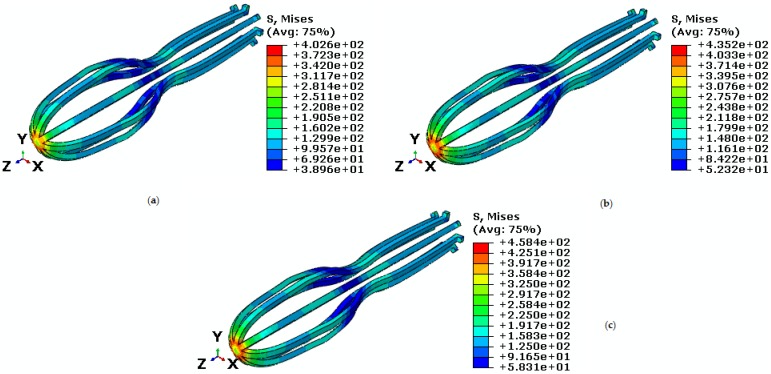
Von Mises stress in the umbrella-shaped implant at temperature of 60 ℃ and tensile forces (**a**) F = 70 N (**b**) F = 80 N (**c**) F = 90 N.

During the process, the pseudoelastic behavior of implant at temperatures higher than $A_{f}$ was studied. To study pseudoelastic properties, the material is in pure austenite phase, which turns into martensite during a loading step and returns to its initial shape (austenite) under the unloading step. Different tensile force levels of 70 N, 80 N, and 90 N in the direction of the z-axis is applied on the implant at the temperature of 60 ℃. Figure 5 shows Von Mises stress under an isothermal condition for comparison between different tensile forces. Our results confirm that critical points of stress are created on the top of the implant as was reported by Yi et al. [7]. Since there is a great tension force at this temperature, the minimum stress is placed on the middle curve of the device. According to Figure 5, the maximum stresses are 402.6 MPa, 435.2 MPa, and 458.4 MPa that are respectively related to the forces 70 N, 80 N, and 90 N.

**Figure 6:**
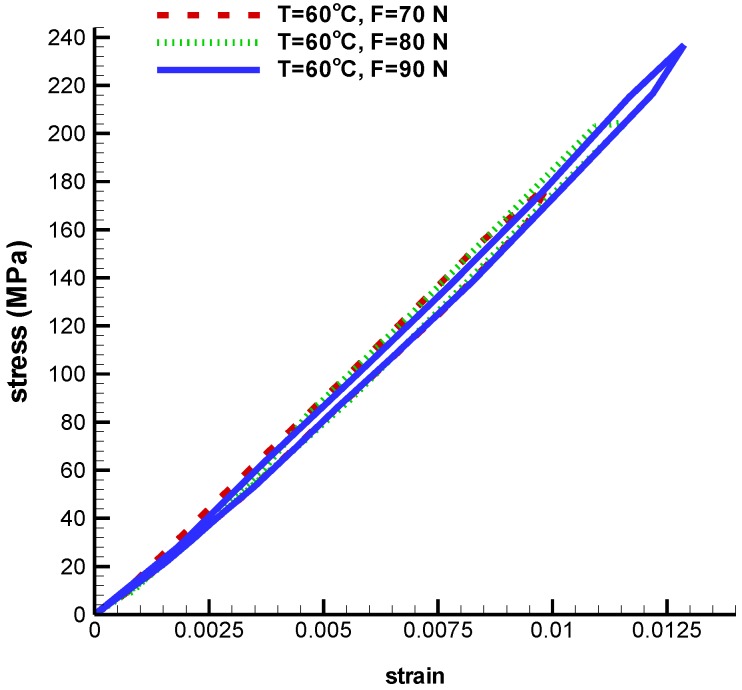
Maximum principal stress-strain diagram (pseudoelastic) of umbrella-shaped implant at temperature of 60 ℃ and tensile forces 70 N, 80 N, and 90 N.

Figure 6 shows a stress-strain curve for three different tensile forces 70 N, 80 N, and 90 N at a constant temperature. As it can be seen, a high-tension force causes higher stress-strain hysteresis loop. According to this figure, the maximum principal strain for tensile forces 70 N, 80 N and 90 N are 0.009, 0.011, and 0.012 respectively.

**Figure 7:**
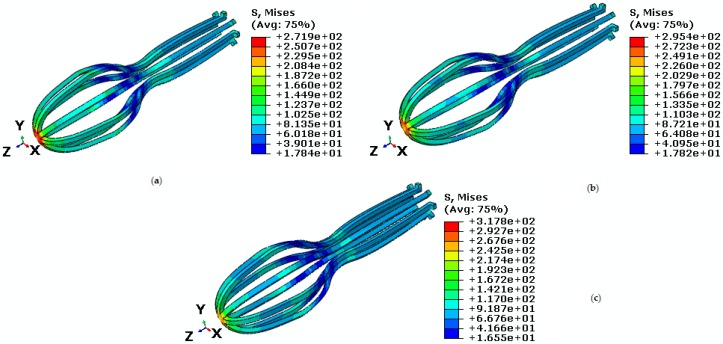
Von Mises stress in the umbrella-shaped implant under 50 N tensile force and (**a**) T = 38 ℃ (**b**) T = 42 ℃ (**c**) T = 60 ℃.

**Figure 8:**
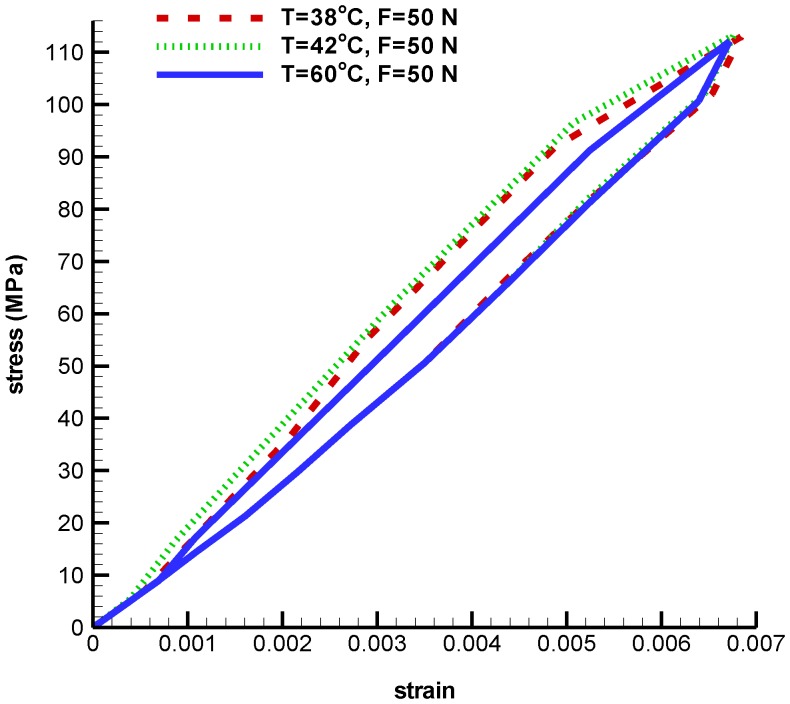
Maximum principal stress-strain diagram (pseudoelastic) of umbrella-shaped implant under 50 N tensile force, 38 ℃, 42 ℃, and 60 ℃ temperatures.

In the following, an umbrella-shaped implant under constant force 50 N and at temperatures 38 ℃, 42 ℃, and 60 ℃ are investigated. According to Figure 7, stress critical points in the middle-upper part of the implant are decreased from 17.8 MP to 16.5 MPa as the temperature increase. Von Mises maximum stress at temperatures 38 ℃, 42 ℃, and 60 ℃ are 271.9 MPa, 295.4 MPa, and 317.8 MPa respectively. For more clarity about pseudoelastic behavior under constant tensile force and different temperature, refer to Figure 8. Since the fever body temperature is 38 ℃, the objective was to study the pseudoelastic of the device performance at this temperature. Maximum principal strain is 0.00683 at temperature 38 ℃, while for temperatures 42 ℃ and 60 ℃ are 0.00676 and 0.00671. Comparisons between maximum principal strains at different temperatures are shown in Table 2. These results show the capability of the simulation process in the prediction of stress distribution on the implant at different conditions.

#### 3.1.2. Pseudoelastic Behavior under Coupling Tension-Torsion 

Here, implant is subjected to tension-torsion force. Three comparisons have been investigated in tension-torsion coupling:

1. The tensile force is variable and torsion torque is constant;
2. The tensile force is constant and torsion torque is variable;
3. Both tensile force and torsion torque are constant and temperature is variable.

**Figure 9:**
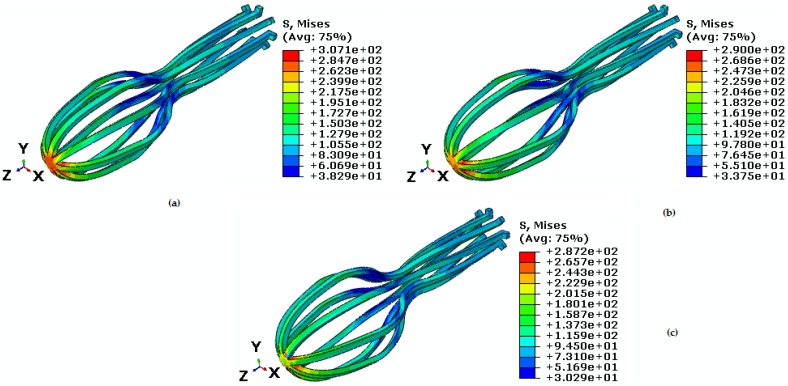
Von Mises stress in the umbrella-shaped implant at 38 ℃ temperature, 75 N·mm torque and tensile forces (**a**) F = 50 N (**b**) F = 40 N (**c**) F = 30 N.

In the first stage, the umbrella-shaped implant is under constant torque, constant temperature, and variable tensile force (Figure 9). In this case, at temperature 38 ℃, torque for the upper part of the umbrella is 75 N·mm and tensile force is variable. As it is shown in Figure 7 and Figure 9, the bulk of the implant is under higher stress when torque is applied. Maximum Von Mises stress at this zone is 271.9 MPa and it is increased to 307.1 MPa when torque is applied. In this case, the Von Mises maximum stress created for forces 50 N, 40 N, and 30 N are 307.1 MPa, 290 MPa, and 287.2 MPa respectively (Figure 9).

**Figure 10:**
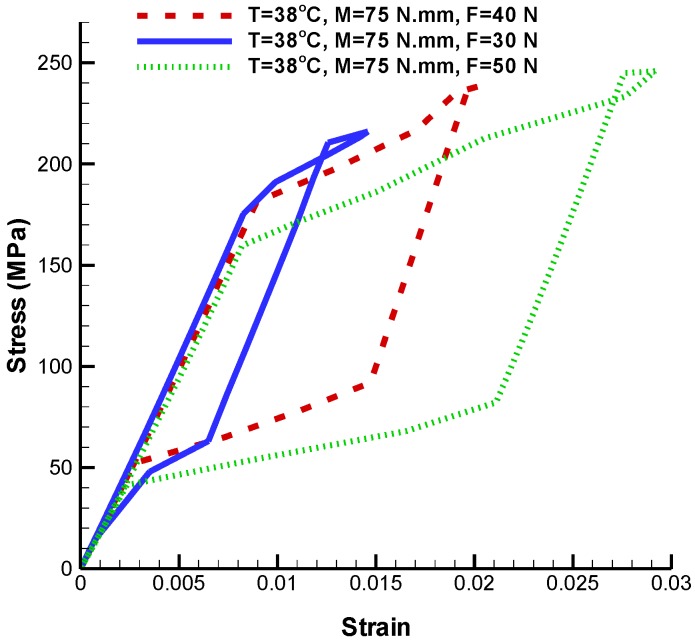
Maximum principal stress-strain diagram (pseudoelastic) of umbrella-shaped implant under 38 ℃ temperature, 75 N·mm torque and 50 N, 40 N, and 30 N tensile forces.

As it is shown in Figure 10, reduction in tensile force makes the stress-strain hysteresis loop smaller at constant torque. The maximum principal strain created in dual loading state for tensile forces 50 N, 40 N, and 30 N are 0.029, 0.02, and 0.014 respectively (Table 3). Consequently, simulations confirm the fact that the umbrella-shaped implant can predict pseudoelastic behavior very well under different loading.

**Figure 11:**
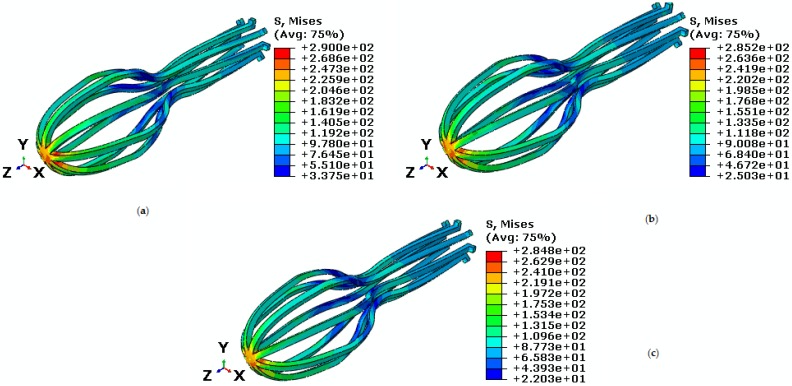
Von Mises stress in the umbrella-shaped implant under 38 ℃ temperature, 40 N tensile force and torques (**a**) M = 75 N·mm (**b**) M = 60 N·mm (**c**) M = 55 N·mm.

During the day, a person may be subjected to different forces. In this section, changes in torque are investigated. Therefore, torque is applied in the different amounts when constant tensile force is applied to the implant at a temperature higher than the body normal temperature. The constant force is 40 N and torque are 75 N·mm, 60 N·mm, and 55 N·mm under temperature 38 ℃. As shown in Figure 11, the highest stresses created are 290 MPa, 285.2 MPa, and 284.8 MPa for torques 75 N·mm, 60 N·mm, and 55 N·mm, respectively.

**Figure 12:**
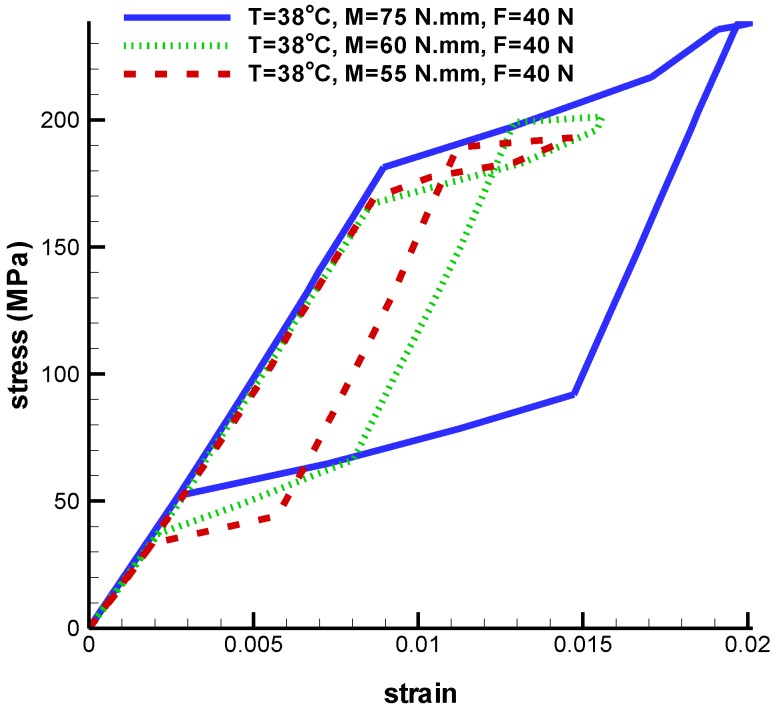
Maximum principal stress-strain diagram (pseudoelastic) of umbrella-shaped implant under 38 ℃ temperature, 40 N tensile force, 75 N·mm, 60 N·mm, and 55 N·mm torques.

Figure 7 and Figure 11 show torque loading will cause critical stress on the umbrella head. To study this matter accurately, refer to Figure 12, in which pseudoelastic property is shown. As shown in this figure, higher torque creates a larger pseudoelastic loop. The maximum strain obtained in the implant for torque values 75 N·mm, 60 N·mm, and 55 N·mm are 0.020, 0.015, and 0.014, respectively (Figure 12).

**Figure 13:**
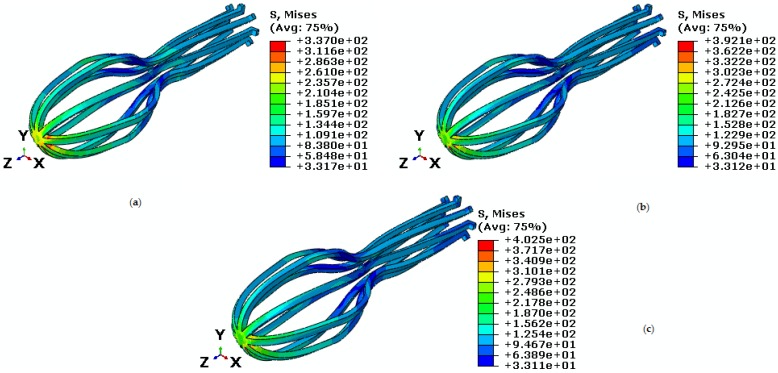
Von Mises stress in the umbrella-shaped implant under tensile force 40 N, torque 75 N·mm and temperatures (**a**) T= 45 ℃ (**b**) T= 50 ℃ (**c**) T= 55 ℃.

**Figure 14:**
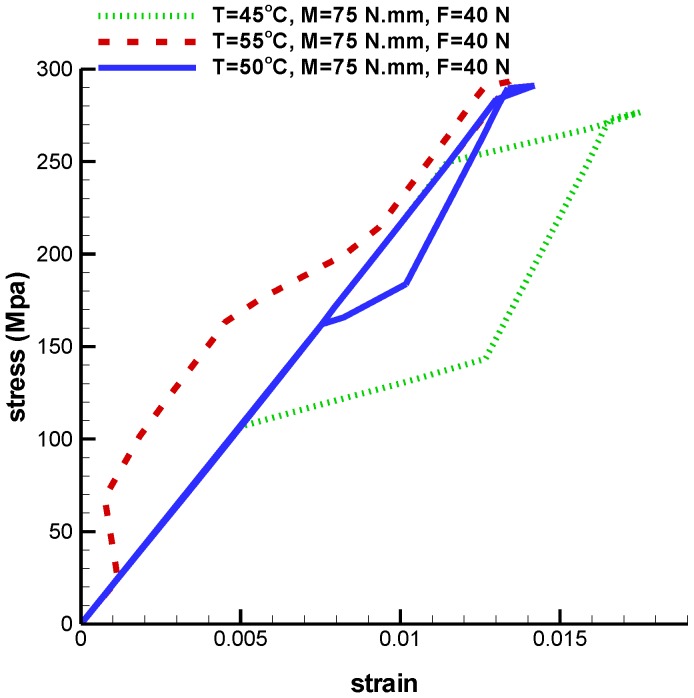
Maximum principal stress-strain diagram (pseudoelastic) of umbrella-shaped implant under 40 N tensile force, 75 N·mm torque and 45 ℃, 50 ℃, 55 ℃ temperatures.

So far, changes regarding force and torque have been investigated at a constant temperature. In the following, a constant force and constant torque are applied to the implant under variable temperatures. At this step, the implant is under 40 N tensile force and 75 N·mm torque at temperatures 45 ℃, 50 ℃, and 55 ℃. According to Figure 13, critical stress in the upper half of the umbrella is increased as temperature increases. The Von Mises maximum stress at temperatures 45 ℃, 50 ℃ and 55 ℃ are 337 MPa, 392.1 MPa, and 402.5 MPa, respectively. Thus, as it is shown in Figure 14, when the temperature goes up, stress increases and consequently strain goes down. As it is shown in Table 3, the maximum principle strain for temperatures 45 ℃, 50 ℃, and 55 ℃ are 0.017, 0.014, and 0.013 respectively.

### 3.2. Study of the Behavior of Umbrella-Shaped Implant under Tension-Torsion Loading at Temperature under $A_{s}$

**Figure 15:**
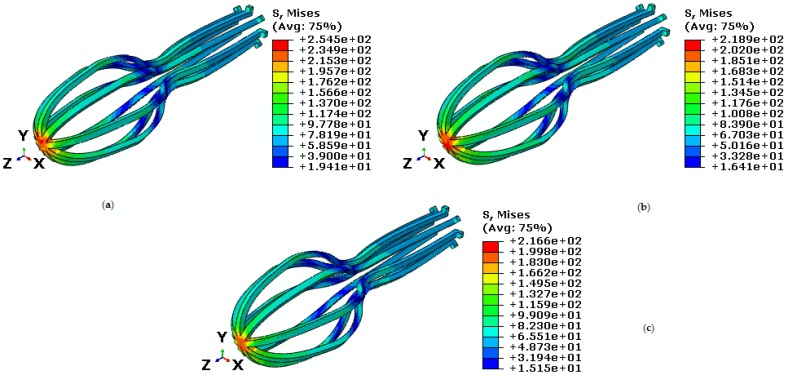
Von Mises stress in the umbrella-shaped implant under 28 ℃ temperature, 30 N·mm torque and tensile force (**a**) F = 42 N (**b**) F = 35 N (**c**) F = 30 N.

**Figure 16:**
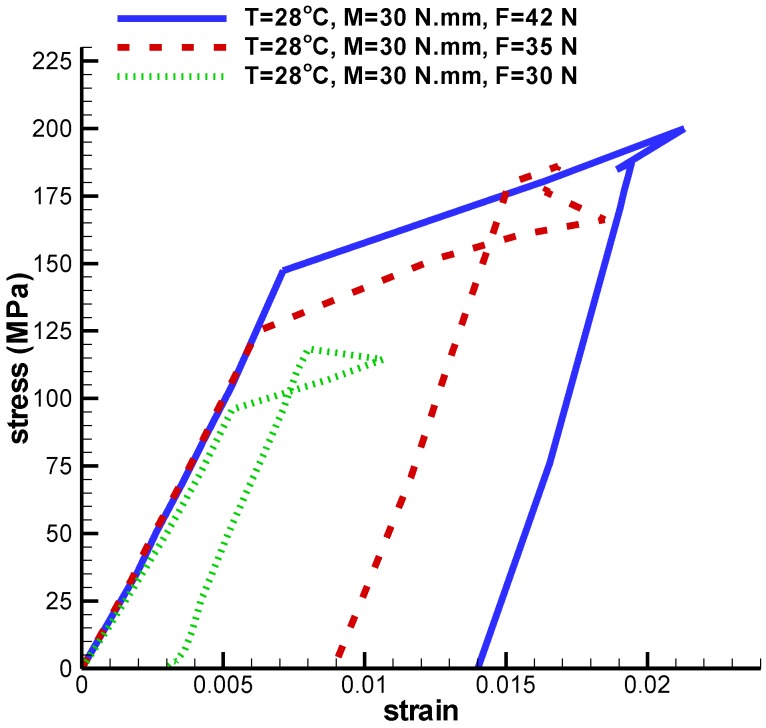
Maximum principal stress-strain diagram (SME) of umbrella-shaped implant under 28 ℃ temperatures, 30 N·mm torque and 42 N, 35 N, 30 N tensile forces.

In this section, the behavior of umbrella-shaped implant at a temperature between $A_{s}$ and $M_{s}$ is investigated. Loading and unloading are applied to the implant, which causes the residual strain reaches to zero if the temperature gets above $A_{f}$. The implant is under tensile force and torque. First, temperature and torque are constant and tensile force is variable. The implant is under the torque 30 N·mm and temperature 28 ℃, and variable tensile forces 42 N, 35 N, and 30 N. The highest Von Mises stresses obtained from these loadings are 254.5 MPa, 218.9 MPa, and 216.6 MPa (Figure 15). Under these loadings, shape memory is well predicted by the implant. Figure 16 shows, increasing axial force will cause higher stress and strain. Therefore, the highest principal strain created for the tensile forces 42 N, 35 N, and 30 N are 0.021, 0.018, and 0.010, respectively (Table 4).

**Figure 17:**
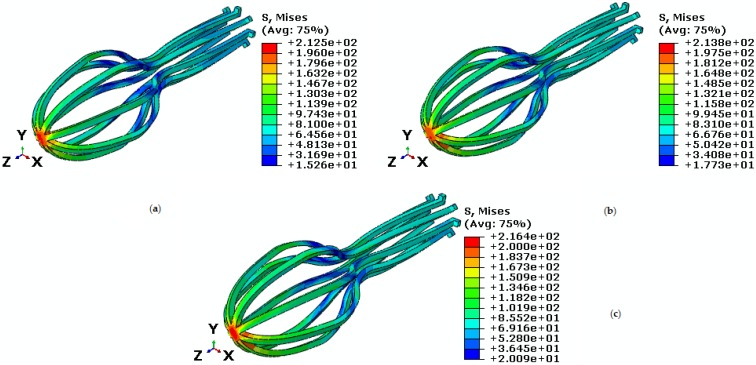
Von Mises stress in the umbrella-shaped implant under 28 ℃ temperature, 30 N tensile force and torques (**a**) M = 40 N·mm (**b**) M = 50 N·mm (**c**) M = 54.5 N·mm.

**Figure 18:**
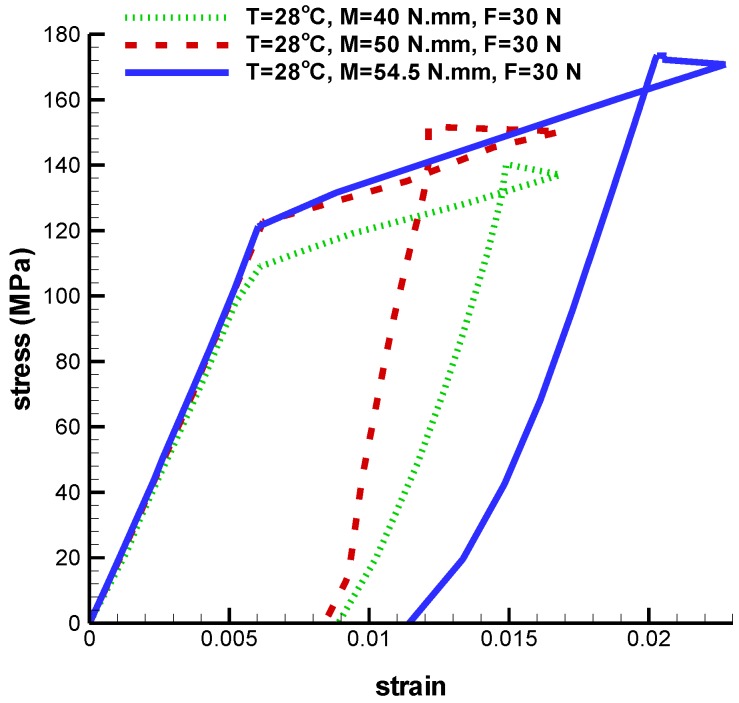
Maximum principal stress-strain diagram (SME) of umbrella-shaped implant under temperatures 28 ℃, tensile force 30 N, torsion torque 40 N·mm, 50 N·mm, and 54.5 N·mm.

Second, force and temperature are kept constant at 30 N and 28 ℃, but torque is variable at 40 N·mm, 50 N·mm, and 54.5 N·mm. For these torques, Von Mises maximum stresses are 212.5 MPa, 213.8 MPa, and 216.4 MPa (Figure 17). In addition, the maximum principal strain at 40 N·mm, 50 N·mm, and 54.5 N·mm torques are 0.01682, 0.01687 and 0.022 respectively (Table 4). Figure 18 shows the SME behavior of implant at different torsion torques.

**Figure 19:**
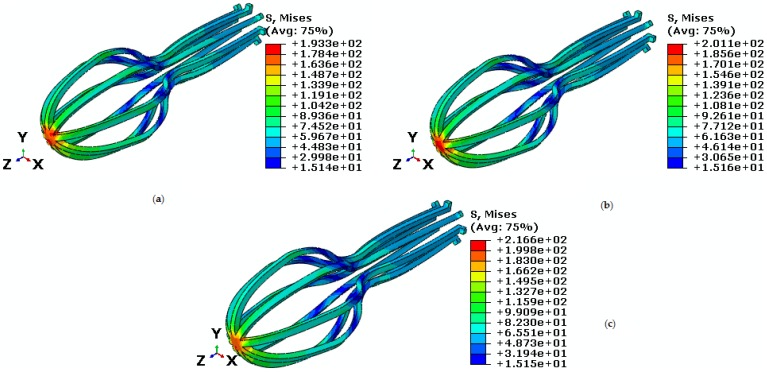
Von Mises stress in the umbrella-shaped implant under tensile force 30 N, torsion torque 30 N·mm and temperatures (**a**) 23.85 ℃ (**b**) 26 ℃ (**c**) 28 ℃.

**Figure 20:**
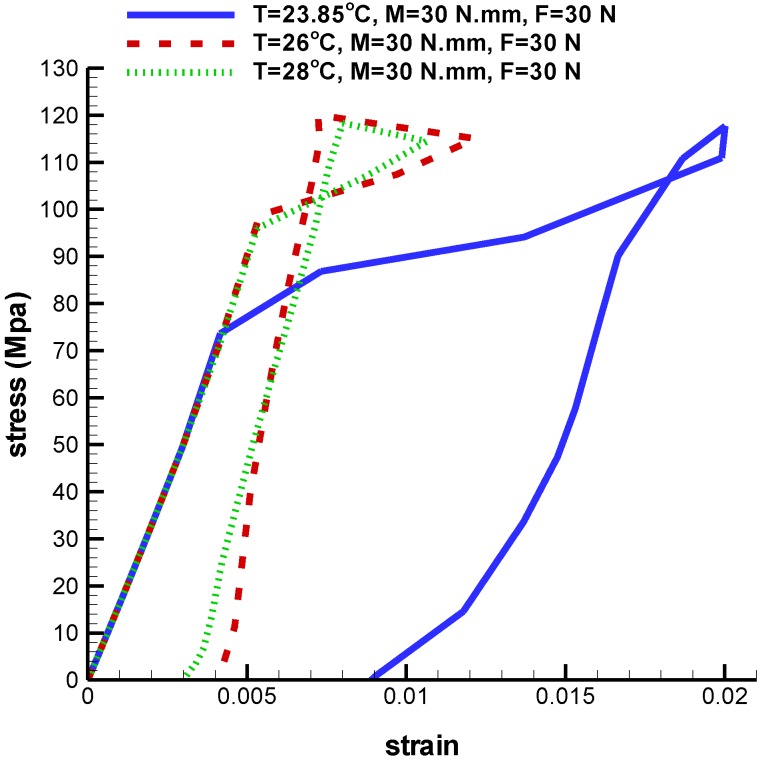
Maximum principle stress-strain diagram (SME) of umbrella-shaped implant under tensile force 30 N, torque 30 N·mm and 23.85 ℃, 26 ℃, 28 ℃ temperatures.

Finally, force and torque are constant while the temperature is variable. As it is seen in Figure 19, the Von Mises maximum stress are 193.3 MPa, 201.1 MPa and 216.6 MPa in temperatures of 23.85 °C, 26 °C, and 28 °C, respectively. As it is shown in Figure 20, when temperature is increased, strain is decreased. The maximum principle strain at temperature of 23.85 °C, 26 °C, and 28 °C are 0.02, 0.012, and 0.010, respectively. Consequently, the results demonstrate that the simulation process is acceptable. Our future study will focus on the investigation of behavior of remaining parts of the femur under real conditions.

## 4. Conclusions 

The objective of this study was to investigate the behavior of NiTi umbrella-shaped implant at two different temperatures under different loadings. To this end, a finite element simulation method is utilized. It can be concluded that the implant has different characteristic under different conditions. At uniaxial loading, the critical areas are located on the top part of the implant. The increase in temperature while the load and torque are constant will cause the strain to decrease from 0.017 to 0.013 at pseudoelastic and from 0.02 to 0.01 at SME regimes. Torque applies more stress at the internal edges of umbrella-shaped implant. The upper part of umbrella-shape implant is under the highest stress during the loading. As a result, when the implant is under a combination of tension-torsion loading, it is likely to break at the bottom portion of the implant. The numerical results show that the least amount of stress is applied to the middle, curved part of the umbrella.

## Figures and Tables

**Table 1:** Shape memory alloy mechanical properties.

Mechanical Properties	Value	Unite
$E_{a}$	26,300	MPa
$E_{m}$	63,000	MPa
$M_{f}$	9	℃
$M_{s}$	18.4	℃
$A_{s}$	29.3	℃
$A_{f}$	37	℃
$C_{M}$	8	MPa/℃
$C_{A}$	13.8	MPa/℃
$\sigma_{s}^{\mathrm{cr}}$	100	MPa
$\sigma_{f}^{\mathrm{cr}}$	170	MPa
$\varepsilon^{*}$	0.067	-

**Table 2:** Von Mises maximum stress and maximum principal strain for different forces and temperatures (pseudoelastic).

Force (N)	Temperature (℃)	Von-Mises Maximum Stress (MPa)	Maximum Principal Strain
70	60	402.6	0.009
80	60	435.2	0.011
90	60	458.4	0.012
50	38	271.9	0.00683
50	42	295.4	0.00676
50	60	317.8	0.00671

**Table 3:** Von Mises maximum stress and maximum principal strain for different force, torque, and temperature (pseudoelastic).

Force (N)	Torque (N·mm)	Temperature (℃)	Von-Mises Maximum Stress (MPa)	Maximum Principal Strain
30	75	38	287.2	0.014
40	75	38	290	0.020
50	75	38	307.1	0.029
40	75	38	290	0.020
40	60	38	285.2	0.015
40	55	38	284.8	0.014
40	75	45	337	0.017
40	75	50	392.1	0.014
40	75	55	402.5	0.013

**Table 4:** Von Mises maximum stress and maximum principal strain for different force, torque and temperature (SME).

Force (N)	Torque (N·mm)	Temperature (℃)	Von-Mises Maximum Stress (MPa)	Maximum Principle Strain
42	30	28	254.5	0.021
35	30	28	218.9	0.018
30	30	28	216.6	0.010
30	40	28	212.5	0.01682
30	50	28	213.8	0.01687
30	54.5	28	216.4	0.022
30	30	23.85	193.3	0.020
30	30	26	201.1	0.012
30	30	28	216.6	0.010
